# Observations on Burns and Scalds

**Published:** 1807-10

**Authors:** 


### *To the Editors of the Medical and Physical Journal.*

Gentlemen,

It never, I believe, can be asserted of any novel discovery, that it was sanctioned by public approbation, "nemine contradicente," (an observation fully confirmed on the the subject of "Burns and Scalds.") Much unnecessary trouble might be spared, if the theorists could but be induced to recollect, that much (as was judiciously hinted by a correspondent in your *last*) "depends on the constitutional variety of the subject," and as much on the different stages or degrees of the accident. If no other inconvenience is sustained than a slight vesication of the injured part, no remedy could be more aptly introduced than the refrigerant application of cold water. But, when the common integuments are so burnt that the cuticle is entirely destroyed, and the parts immediately affected with great pain and vesication, the sedative effects of cold Ablution would be highly improper, and the stimulant qualities of the terebinthinate application much preferable.

I am fully convinced that any discerning mind, open to conviction and unbiassed by prejudice, will readily perceive the impropriety of using sedative applications in constitutions, where, from natural inactivity, every thing stimulating is required, in order to counteract the inanition of the system. It is far from my desire or intention to take from Dr. Kinglake's valuable discovery, any of the credit to which it is deservedly entitled; on the contrary, whenever the general tenor of the constitution will admit of topical refrigeration, there is no treatment, I conceive, so well calculated to lessen the pain of the patient, and diminish the perplexity of the practitioner, as cold ablution. And it is to be hoped, that Dr. Kentish will rest satisfied, that *his* plan of treatment is considered *equally* efficacious with that of Dr. Kinglake, provided they are *alternately* and *judiciously* adopted.

Various other modes of treatment have been recommended for the cure of this accident; while some treat it as a species of inflammation, others recommend the injured part to be applied as near the fire as the patient can bear, and repeat it while any sense of heat or pain remains, upon a supposition that, by this method, the stagnating fluids are repelled into their proper channels, and the vesication, or other consequences, thereby prevented. It should seem, however, sufficient to confine ourselves to the two former applications of heat and cold, being convinced that the *respective* principles upon which they act, are as rational as the relief they afford is permanent.

I am, &c.

MEDIUM.

